# Gastric Proteins MUC5AC and TFF1 as Potential Diagnostic Markers of Colonic Sessile Serrated Adenomas/Polyps

**DOI:** 10.1093/ajcp/aqw142

**Published:** 2016-10-18

**Authors:** Magomed Khaidakov, Keith K. Lai, D. Roudachevski, Julietta Sargsyan, Hannah E. Goyne, Rish K. Pai, Laura W. Lamps, Curt H. Hagedorn

**Affiliations:** From the 1 Department of Medicine; 2 Department of Pathology, University of Arkansas for Medical Sciences, Little Rock;; 3 Central Arkansas Veterans Healthcare System, Little Rock;; 4 Department of Pathology, Mayo Clinic, Scottsdale, AZ.

**Keywords:** Serrated polyps, Sessile serrated adenomas/polyps, Hyperplastic polyps, MUC5AC, TFF1, Colon cancer

## Abstract

**Objectives:** A subset of colon cancers originates from sessile serrated adenomas/polyps (SSA/Ps). Our goal was to identify markers for SSA/Ps that could aid in distinguishing them from hyperplastic polyps (HPs).

**Methods:** We performed immunostaining for gastric proteins MUC5AC and TFF1 in formalin-fixed, paraffin-embedded (FFPE) samples of HPs (n = 47), SSA/Ps (n = 37), and normal colon (n = 30).

**Results:** Control mucosa expressed only trace amounts of MUC5AC and TFF1. HPs exhibited an 11.3- and 11.4-fold increase in MUC5AC and TFF1 expression confined to the upper segments of the crypts near the luminal surface of the polyps. SSA/Ps displayed on average 1.6-fold (MUC5AC, *P* < .008) and 1.4-fold (TFF1, *P* < .03) higher signal intensity for these markers than HPs, with a dramatic coexpression of MUC5AC and TFF1 typically occupying the entire length of the crypt. Immunoperoxidase results were similar to immunofluorescence staining for both MUC5AC and TFF1.

**Conclusions:** Our results suggest that the analysis of expression of MUC5AC and TFF1 may be useful for differentiating SSA/Ps from HPs. We also suggest the possibility that crypt morphology may be at least partly due to overproduction of highly viscous gastric mucins and that these proteins may play a role in the serrated pathway to colon carcinogenesis.

Colon cancer is the second largest cause of cancer-related deaths in the United States.^1^ Colonic neoplasms originate primarily from colon polyps and develop via partially overlapping but mechanistically distinct pathways that have been designated as the adenomatous and serrated pathways. About 60% of colon cancers are thought to originate from adenomas via “suppressor” or “mutator” pathways, involving *APC* mutations in combination with constitutive stimulation of RAS-RAF-MAPK signaling due to gain-of-function mutations in *BRAF* or *KRAS.*^2^^,^^3^ Accumulating evidence indicates that most other colon adenocarcinomas, possibly 20% to 30%, arise from a subset of serrated polyps, designated sessile serrated adenomas/polyps (SSA/Ps), which were previously classified as hyperplastic polyps and thought to have little or no tumorigenic potential.^4‐7^

The “serrated pathway” is believed to be responsible for the progression of SSA/Ps to colonic adenocarcinoma.^2^^,^^8^ In addition, SSA/Ps exhibit a high frequency of gain-of-function mutations in the *BRAF* gene, ranging from 70% to 100%^9‐13^; *BRAF* mutations have been implicated in a variety of cancers due to their activation of the MAPK signaling pathway.^14^

The current pathologic classification of serrated polyps includes hyperplastic polyps (HPs), traditional serrated adenomas (TSAs), and SSA/Ps,^8^^,^^15^ with the latter displaying the strongest association with an increased risk for colon cancer. SSA/Ps are histologically distinct from HPs. They typically exhibit full-length serration of crypts in combination with lateral dilatation or “boot”-shaped deformities at the crypt bases as well as “reverse maturation,” or the presence of mature goblet cells and/or foveolar-type cells at the base.^8^

Nevertheless, differentiating between SSA/Ps and HPs on routine histologic examination can be challenging, particularly in small or fragmented samples. This has been highlighted by a number of studies documenting the frequent misclassification of SSA/Ps as HPs,^16^^,^^17^ resulting in inadequate follow-up. Conversely, misclassifying an HP as an SSA/P may result in unnecessary cancer screening in these patients. A reliable diagnostic test that may help in this distinction would be very useful in identifying SSA/Ps so that appropriate follow-up and screening could be provided to the large number of patients with serrated polyps.

In our previous studies,^13^ we used RNA sequencing in evaluating the transcriptional signature of syndromic SSA/Ps with the purpose of identifying highly differentially expressed genes that could be candidate diagnostic markers for SSA/Ps. A significant fraction of the genes that were overexpressed more than 10-fold in SSA/P coded for extracellular and membrane proteins involved in mucus formation and maintenance. Interestingly, the transcriptional signature of SSA/Ps included genes that are abundantly expressed in gastric epithelium, such as *MUC5AC* and *TFF1*, but not normally expressed in colonic mucosa. In the present study, we evaluated the expression of MUC5AC and TFF1 by immunofluorescence and immunoperoxidase staining of SSA/Ps and HPs to determine their possible suitability as diagnostic markers for SSA/Ps.

## Materials and Methods

### Samples and Pathologic Examination

A total of 37 SSA/Ps, 46 HPs (41 microvesicular and five goblet cell), and 30 normal colons were retrieved from the archives of the University of Arkansas for Medical Sciences and Central Arkansas Veteran Healthcare Center System. All specimens were biopsy samples obtained by colonoscopy and were formalin-fixed and paraffin embedded (FFPE). All specimens were reviewed and the diagnoses confirmed by two pathologists with expertise in gastrointestinal (GI) pathology (K.K.L. and L.W.L.). Because this is the initial study in determining the utility of these markers, we limited our analysis to morphologically unequivocal^8^^,^^13^ HPs from the left colon and SSA/Ps from the right (splenic flexure to cecum) colon.

### Immunofluorescence and Immunohistochemistry

Antibodies for MUC5AC (cat. MA512178) and TFF1 (cat. PA128875) were purchased from Fisher Scientific (Hanover Park, IL). For immunofluorescence staining, 4-μm sections of FFPE were mounted on positively charged Superfrost/Plus slides (ThermoFisher Scientific, Grand Island, NY). Sections were deparaffinized with xylene and rehydrated using graded series of alcohol to phosphate-buffered saline (PBS). Antigen retrieval was performed by incubating slides in 10 mM citrate buffer (pH 6.0) in a water bath at 95 °C for 30 minutes, followed by incubation at room temperature (RT) for another 30 minutes. Tissue sections were treated with blocking buffer (1% bovine serum albumin [BSA], 0.012% saponin in PBS) for 30 minutes, incubated with primary antibodies (1:100 dilution in blocking buffer) for 2 hours at RT in a humidity chamber, and washed in PBS-PBS with 0.5% Tween-20 (PBST)-PBST (10 minutes each). Immunofluorescence analysis samples were incubated with the appropriate DyLight-conjugated secondary antibody (Molecular Probes, Grand Island, NY) at dilutions of 1:1,000 for 1 hour at 37 °C in a humidity chamber following the manufacturer’s recommendations. After three consecutive 10-minute washes with PBS, PBST and PBS coverslips were mounted using Prolong Diamond antifade mountant with 4′,6-diamidino-2-phenylindole (Molecular Probes) and imaged by fluorescent microscopy. For peroxidase immunohistochemistry (IHC) analysis, deparaffinized 4-μm sections were preincubated with a 2.5% normal horse serum blocking solution (cat. S-2012; Vector Laboratories, Burlingame, CA) for 30 minutes at RT and incubated with primary antibodies for 1 hour at RT. Samples were washed with PBS and PBS with 1% Tween 20. Peroxidase immunostaining was performed, after treatment with BLOXALL (Vector Laboratories) endogenous peroxidase blocking solution, using the ImmPRESS polymer system and ImmPACT DAB substrate (Vector Laboratories) per the manufacturer’s instructions. Controls included no primary antibody.

### Image Analysis

Immunofluorescence image analysis was performed using ImageJ software (National Institutes of Health, Bethesda, MD). The mean intensities of signals on RGB split images corrected for background were measured in at least three representative crypts per sample and averaged. To quantify the number of cells with sufficient colocalization, we used a program, “Intensity Correlation Analysis,” located within the colocalization plugins for ImageJ, to generate gradient intensive images. The number of cells was counted from each subsequent image: MUC5AC (green), TFF1 (red), and colocalization (yellow). The coexpression was determined as a percentage of cells with colocalized signals for MUC5AC and TFF1. In addition to software-based analysis, expression and colocalization were scored by two GI pathologists (K.K.L. and H.E.G., reviewed by L.W.L.) based on the percentage of serrated crypt cells staining (0, none; 1, 1%-25%; 2, 26%-50%; 3, 51%-75%; 4, >76%) and intensity of staining (0-4).

### Statistical Analysis

Data are presented as mean ± SD. The statistical analysis was performed with SPSS 11.5 software (SPSS, Chicago, IL). Multiple comparisons were analyzed by one-way analysis of variance (ANOVA). A *P* value less than .05 was considered significant.

## Results

### MUC5AC and TFF1 Expression in HPs and SSA/Ps

**Image 1:**
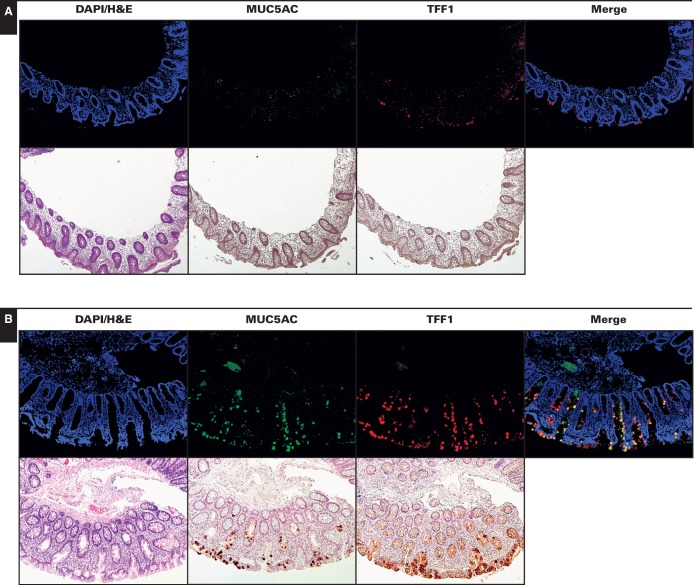

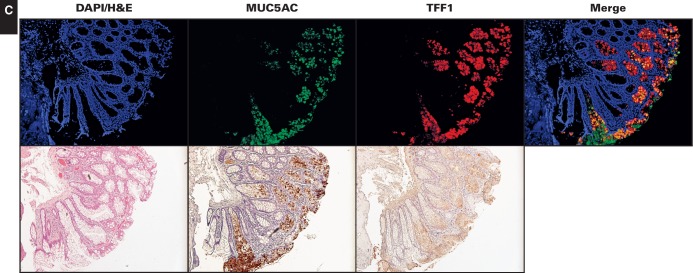
Expression of MUC5AC and TFF1 in normal colon (**A**), hyperplastic polyps (HPs) (**B**), and sessile serrated adenomas/polyps (SSA/Ps) (**C**) (x10). Immunofluorescence and immunoperoxidase staining of the same region is shown for representative control (normal), HP, and SSA/P samples. As expected, the signals for gastric proteins MUC5AC and TFF1 were negligible in normal colonic mucosa. MUC5AC and TFF1 immunopositive cells were present in HPs. In SSA/Ps, MUC5AC and TFF1 were typically expressed by most of the epithelial cells along the entire length of architecturally compromised crypts. SSA/Ps, compared with HPs, also showed significant coexpression of both MUC5AC and TFF1 in merged immunofluorescence analyses.

**Figure 1:**
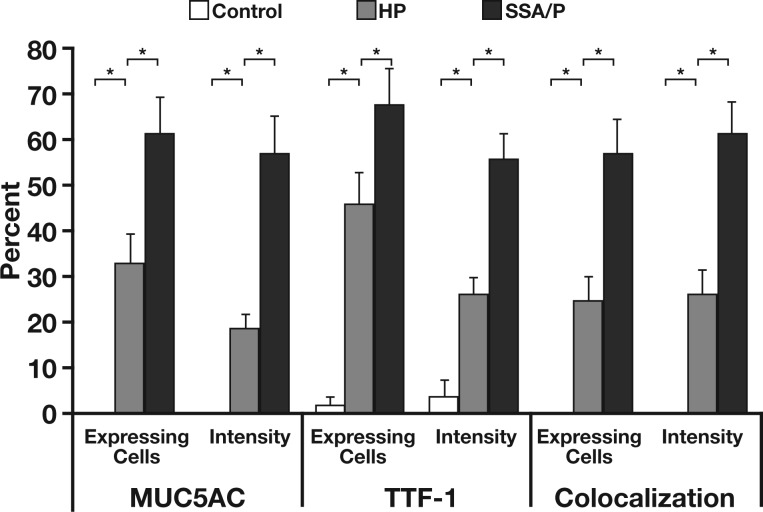
Bar graphs illustrating the differences in immunofluorescence signal intensities for MUC5AC and TFF1 in normal colon, hyperplastic polyps, and sessile serrated adenomas/polyps. The images were scored by experienced gastrointestinal pathologists (K.K.L. and H.E.G.) based on the percentage of serrated crypt cells with immunostaining (0, none; 1, 1%-25%; 2, 26%-50%; 3, 51%-75%; 4, >76%) and intensity of staining (0-4). *P < .02, two-tailed.

With the exception of rare epithelial cells located near the luminal surface that expressed TFF1 or MUC5AC **Image 1**, all 30 normal colon controls had negligible staining for MUC5AC and TFF1. This is in agreement with prior RNA sequencing gene expression studies that did not show expression of these proteins in the normal colon.^13^ In contrast, HPs exhibited immunopositivity for both MUC5AC and TFF1 (11.3-fold vs control, *P* < 10^−^^6^ and 11.4-fold vs control, *P* < 10^−^^6^, respectively; see Image Analysis) (Image 1 and **Figure 1**). No major difference was observed between the staining patterns of goblet cell (n = 5) and microvesicular (n = 41) HPs. SSA/Ps displayed the highest expression of MUC5AC and TFF1 by immunostaining (17.5-fold vs control, *P* < 10^−^^9^ for MUC5AC and 16.3-fold vs control, *P* < 10^−^^9^ for TFF1), which often involved the entire length of the crypt, including the base. The differences in staining between SSA/Ps and HPs for both proteins were statistically significant (*P* < .008 for MUC5AC and *P* < .03 for TFF1). Immunoperoxidase staining of MUC5AC and TFF1 in SSA/Ps and HPs showed similar results to immunofluorescence staining (Image 1).

### Compared With HPs, SSA/Ps Exhibit Stronger Colocalization of MUC5AC and TFF1

**Image 2:**
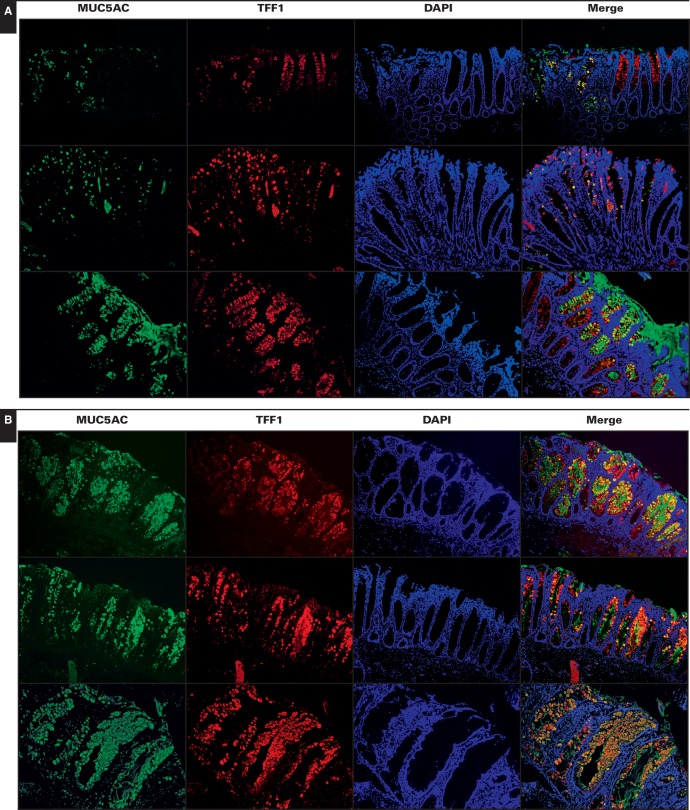
Representative examples of colocalization of MUC5AC and TFF1 in different hyperplastic polyps (HPs) (**A**) and sessile serrated adenomas/polyps (SSA/Ps) (**B**) (x20). Compared with HPs, most SSA/P samples showed significant colocalization often approaching 100%. HPs also exhibited greater variability in the distribution and colocalization of signals. Expression of MUC5AC and TFF1 was generally observed in separate cells with little colocalization. Two of 46 HPs analyzed demonstrated more coexpression of MUC5AC and TFF1 suggestive of but not identical to SSA/Ps (**A**, bottom row).

In terms of colocalized immunostaining for MUC5AC and TFF1, HPs showed heterogeneous staining with a range of phenotypes **Image 2A**. Most HPs showed some cells with colocalization of MUC5AC and TFF1 (Image 2A, top two rows), with two exceptions of 46 HPs analyzed showing more colocalization (Image 2A, bottom row). In contrast, SSA/Ps almost uniformly demonstrated strong, intense colocalization of MUC5AC and TFF1 immunostaining **Image 2B**. Based on scoring by GI pathologists (see Materials and Methods), the degree of colocalization of MUC5AC and TFF1 reached roughly 25% in HPs, whereas colocalization in SSA/P samples approached 57% (*P* < .008) (Figure 1). Regardless of the degree of colocalization, the expressions of MUC5AC and TFF1 significantly correlated (*r*^2^ = 0.48, *P* = .0007).

## Discussion

Due to the inherent difficulties in distinguishing between HPs and SSP/As that are encountered in the routine practice of pathology, identifying clinically applicable biomarkers that distinguish between SSA/Ps and HPs has been an area of ongoing research for some time. In this study, we have shown that MUC5AC and TFF1, genes commonly expressed in gastric mucosa, are found in both HPs and SSA/Ps but differ in their distribution and coexpression. Although additional larger studies are needed, the different expression properties of MUC5AC and TFF1 in SSA/Ps and HPs may serve as clinically useful diagnostic criteria.

MUC5AC belongs to a family of secreted mucins found primarily in the mucosa of the respiratory tract, stomach, and reproductive organs.^18^ Deregulation of MUC5AC is implicated in a number of nonneoplastic diseases, including cystic fibrosis,^19^ chronic obstructive pulmonary disease,^20^ asthma,^21^ inflammatory bowel disease,^22^ and a variety of cancers.^23^^,^^24^ Notably, the appearance of MUC5AC in epithelial cells that do not normally produce and secrete MUC5AC is a common hallmark of malignant progression. Neoplasms originating from a wide variety of epithelial tissues, including colon, overexpress MUC5AC, yet there is little or no expression in the corresponding normal tissue.^25^

Trefoil factors are a three-member family of small secreted proteins involved in the repair of mucosal damage due to their ability to inhibit apoptosis and stimulate cell migration, proliferation, and angiogenesis.^26^^,^^27^ Similar to MUC5AC, TFF1 is primarily expressed in gastric mucosa.^28^ Individual trefoil factors have been also noted to preferentially interact with specific mucins, and TFF1 has been shown to coexpress with MUC5AC in gastric epithelium.^29^ Depending on the location and other factors, TFF1 may act as either a tumor suppressor or pro-oncogene. In gastric mucosa, which is characterized by high basal expression of TFF1, malignant progression is associated with its loss, in part, due to hypermethylation.^30^^,^^31^ Conversely, TFF1 expression is increased in premalignant lesions of the breast and pancreas.^32^

MUC5AC and TFF1 exemplify the double-edged nature of mucosal defense-repair mechanisms. Both proteins are part of signaling pathways associated with tissue repair and carcinogenesis. As a part of the chromosome 11p15 cluster of secreted mucins, MUC5AC can be transcriptionally stimulated in parallel with MUC2, MUC5B, and MUC6.^33^ The mechanisms of upregulation of MUC5AC in cancers include promoter hypomethylation, inflammatory interleukins, TGFβ/Smad4, HIF1α, COX2, and GLI1.^34‐39^ TFF1 has been shown to be regulated by GATA6 in the colon^40^ and is a target gene for estrogen receptor–mediated signaling in mammary epithelium.^41^ There is also a significant overlap in the regulation of MUC5AC and TFF1. Transcription of both MUC5AC and TFF1 is also modulated by MAPK signaling, which is activated by extracellular signals such as growth factors and inflammatory cytokines.^42^^,^^43^ Proposed cancer progression roles for MUC5AC in adenocarcinoma include overexpression of MUC5AC in intercellular junctions that interfere with the membrane localization of E-cadherin, compromise cell-cell adhesion, and increase migration and invasion of pancreatic ductal adenocarcinoma cells.^39^ MUC5AC may also promote carcinogenesis by shielding cells from immune surveillance.^44^ Forced expression of TFF1 in colonic adenoma cells provokes anchorage-independent growth and increased growth of xenografts.^45^ Similarly, overexpression of TFF1 stimulates motility or metastasis of human pancreatic stellate or carcinoma cells.^32^ Some of these effects may be attributed to TFF1-mediated transcriptional inhibition of E-cadherin.^46^ Another possible mechanism involves transactivation of epidermal growth factor receptors.^47^

The present study revealed significant differences in expression of MUC5AC and TFF1 in SSA/Ps and HPs. Our data corroborate previously reported observations on aberrant overexpression of these proteins in serrated colonic polyps.^48‐51^ In previous studies, however, hyperplastic polyps were not yet differentiated according to modern classification,^48^^,^^49^ or the analysis did not focus on potential use of gastric proteins as diagnostic markers of SSA/Ps.^50^^,^^51^ We observed a significant increase in not only the intensity of immunostaining but also the percentage of cells expressing MUC5AC and TFF1 in SSA/Ps.

A recent study of 722 colorectal carcinomas determined that protein expression of MUC5AC, MUC2, and MUC6 was strongly associated with CIMP, V600E *BRAF* mutations, poor differentiation, and increased T stage and inversely associated with p53 expression.^52^ Based on both the literature and daily clinical practice, pathologists are aware that many serrated polyps cannot be reliably classified based on morphology alone, which in turn affects the clinician’s ability to assign appropriate follow-up to many patients with serrated polyps.^16^^,^^17^ Our findings suggest that TFF1 and MUC5AC are capable of distinguishing potentially tumorigenic polyps within the serrated polyp and could aid in the distinction between SSA/P and HP in histologically suboptimal samples.
